# Histopathological characteristics of metastasizing squamous cell carcinoma of the skin and lips

**DOI:** 10.1111/j.1365-2559.2006.02472.x

**Published:** 2006-02-09

**Authors:** P J F Quaedvlieg, D H K V Creytens, G G Epping, C J Peutz-Kootstra, F H M Nieman, M R T M Thissen, G A Krekels

**Affiliations:** 1 Department of Dermatology and Department of Pathology, University Hospital Maastricht Maastricht; 2 Department of Dermatology and Department of Dermatology, Catharina Hospital Eindhoven Eindhoven; 3 Eindhoven and Department of Clinical Epidemiology and Medical Technology Assessment, University Hospital Maastricht Maastricht, the Netherlands

**Keywords:** histopathology, metastasis, squamous cell carcinoma

## Abstract

Quaedvlieg P J F, Creytens D H K V, Epping G G, Peutz-Kootstra C J, Nieman F H M, Thissen M R T M & Krekels G A (2006) *Histopathology* **49**, 256–264

**Histopathological characteristics of metastasizing squamous cell carcinoma of the skin and lips**

**Aims:**

The reported incidence of metastasis from squamous cell carcinoma (SCC) of the skin and lip varies between 0.5% and 16%. Clinical and histopathological criteria have been proposed to identify tumours that may have an increased risk of metastasis. The aim of this study was to define such high-risk tumours, especially since the incidence of SCC of the skin is increasing.

**Methods and results:**

Histopathological features of metastasized skin and lip tumours and a matched group of non-metastasizing tumours were reassessed. Characteristics studied were: tumour width, excision margins, histological subtype, Clark level, Breslow depth, tumour differentiation, inflammation, perineural and angio-invasive growth, ulceration and desmoplasia. Data were statistically analysed separately for skin and labial lesions. Desmoplasia, Clark level, Breslow depth, maximum diameter, angio-invasion, grading, perineural invasion, plasma cells and eosinophilic inflammatory response proved to be statistically significantly related to metastasis of skin tumours. Breslow depth, plasma cells and grading appeared to be statistically significantly related to metastasis of SCC of the lips.

**Conclusions:**

A typical metastatic SCC showed: a tumour width of at least 15 mm, a vertical tumour thickness (= Breslow) of at least 2 mm, less differentiation, presence of desmoplasia and an inflammatory response with eosinophils and plasma cells.

## Introduction

Squamous cell carcinoma (SCC) of the skin is the second most frequent malignant tumour in the head and neck region and is associated with an appreciable rate of metastasis (0.5–16%).^1^–^10^ The registration of SCCs is inadequate; many patients are treated in private practice and excised tumours sometimes have no histological verification. The potential for metastatic disease with SCC of the skin is often under-emphasized, especially among dermatologists who frequently handle malignant degeneration of actinic lesions.

The potential for metastasis may also be underestimated because patients presenting with tumour in a lymph node may not associate such metastasis with a skin cancer treated several years previously. At present, it is unpredictable whether a SCC will metastasize or not.

The clinical classification of SCC, as established primarily by the International Union against Cancer (UICC), does not allow an optimal estimation of metastatic risk. The TNM (Tumor Node Metastases) staging system has proved satisfactory for many malignant tumours, but is not reliable for assessment of the metastatic potential of skin SCC; clinical experience shows that even small carcinomas can metastasize. The question therefore arises which additional prognostic factors,^11^ such as anatomical site, histological features, aetiology, host immunosuppression and prior treatment, are indicative of the metastatic risk of a tumour.^12^–^14^

Petter, Haustein and Breuniger,^12^–^17^ found that tumour size, Clark level, Breslow depth, mitotic index, ulceration, blood vessel invasion, lymphatic invasion, tumour-associated tissue eosinophilia and desmoplasia are related to metastasis. In our study we investigated what proportion of SCCs metastasize and if histopathological features can be identified to predict metastatic potential.

The prevalence of metastasis was determined for two locations of the tumour, the lips and other locations within the skin, because SCC of the lip shows a higher risk of metastasis.^8^

## Materials and methods

The PALGA registry system (a national registry system of all histopathological specimens in the Netherlands) was used to obtain the records of all registered SCCs of the skin and metastases in the University Hospital of Maastricht in the period between 1982 and 2002.

All available tissue sections of the tumours that metastasized were compared with a control group of non-metastasizing tumours. Both groups were reassessed by both dermatologist and dermatopathologist for histopathological characteristics. We matched tumours for the following factors: gender, primary or recurrent lesion, use of immunosuppression, location of the lesion, prior therapy and the length of the follow-up period. Recurrent lesions were defined as lesions with the same histology that showed complete clinical response to treatment, but reappeared at the same site. Information regarding immune status was obtained from electronic patient files. The follow-up period was defined as the time from the diagnosis to the beginning of our study (January 2003). To diminish the possibility of missing metastases in the matched group, we used matched cases with a follow-up of at least 3 years because SCC tumours usually metastasize after 3 years or longer. The primary outcome variable of this study was metastasis, scored as 0 (absent) or 1 (present).

The tumour characteristics assumed to be related to metastasis based on previous studies were: tumour width, excision margins, histological subtype, Clark's depth, Breslow depth, tumour differentiation, inflammatory cell invasion (lymphocytes, eosinophils, neutrophils, histiocytes, plasma cells), perineural and angio-invasion, ulceration and desmoplasia.

The tumour width was taken from the histology reports and patient files. In most cases the exact tumour size was recorded in these reports. In some cases, however, no size was mentioned. In those cases the maximum size of the skin excision was used corrected for the tumour-free margin. When there was no reporting of margins, we assumed that there was a clinical tumour-free margin of 5 mm, which is the clinical standard in our hospital. Excision margins were stated free with wide margins, i.e. > 2 mm (1), involved (2) or free with narrow margins (3). Bracketed numbers represent the score given to the respective parameters. The SCC was subtyped as being a regular horn forming SCC (1) or one of the less common forms of SCC, being: acantholytic SCC (2), spindle cell SCC (3), basosquamous SCC (4) desmoplastic SCC (5) or verrucous SCC (6). Clark's depth was scored according to the anatomical invasiveness, based on Clark's scoring system. The most invasive point was measured.

Breslow depth was measured from the granular layer of the skin to the deepest point with an accuracy of 0.1 mm. Tumour differentiation was classified into well (1), moderately (2) or poorly (3) differentiated. Grading was based on cellular pleomorphism and (de)squamation.

The inflammation (lymphocytes, histiocytes, eosinophils, neutrophils, plasma cells) in the tissue was subtyped and scored dichotomously. No or limited invasion with inflammatory cells was scored as 0, moderate and major invasion was scored 1.

Perineural invasion and angio-invasion were scored after staining. Slides were stained for perineural invasion using staining for pankeratins (MNF-116) and S100 antigen. Angio-invasion was identified using immunohistochemistry for CD31. Invasion was recorded as either absent (0) or present (1). Ulceration and desmoplastic stromal reaction were graded using the same two-point scale as for inflammatory cell infiltrate.

### Statistics

Data were analysed separately for skin and lip tumours. In both analyses, having or not having a metastasis was considered to be the outcome variable. If metric variables (or variates) appeared to be non-normally distributed and were regarded as an outcome variable, an attempt was made to normalize their distribution by transforming them by logarithms (based on 10).

Student's *t*-test was also performed. Tumour characteristics considered to be relevant for prediction of the prevalence of metastasis consisted of a set of two continuous variables and 13 discrete classifications. First, log-likelihood χ^2^ and odds ratios [with 95% confidence intervals (CIs)] were calculated for each separate risk (or protective) variable or factor. For this purpose continuous variables were also dichotomized on their median value. Next, a multivariate logistic regression analysis was carried out using the backward elimination log-likelihood χ^2^ technique with all relevant variables and factors. The result of this search technique for the best fitting model is presented as a reduced logistic regression model containing only statistically significant predictors. Net-effect odds ratios (and 95% CIs) of this eventual model are presented in Table 1. A *P*-value < 0.05 was considered to be statistically significant. All data analysis was performed with SPSS-pc version 12.0 (SPSS Inc., Chicago, IL, USA).

**Table 1a:** Skin histopathological characteristics of the skin related to metastasis (*n* = 110)

		*n*	%META'S	X^2^L	*P*	OR	Lower	Upper	*n*
Grading	1	41	51			2.48	1.03	5.94	
	2	41	51			2.48	1.03	5.94	
	3	22	86			14.93	3.8	58.68	
Clark*	1	19	0	51.38	<0.001	–	–	–	
	2	11	9			5.57	0.21	149.16	
3	28	46			33.95	1.87	617.08	
4	27	70			89.48	4.83	1659.05	
5	21	86			206.23	9.95	4268.42	
RClark	1 + 2 + 3	58	24	32.65	<0.001	–	–	–	106
	4	27	70			7.46	2.69	20.74	
	5	21	86			18.86	4.83	73.63	
Max diam	≤15	51	24	26.97	<0.001	9.25	3.74	22.85	101
	>15	50	74						
Breslow	≤2.85	45	13	42.90	<0.001	25.28	8.17	78.20	89
	>2.85	44	80						
Perineural invasion	0	64	45	14.97	<0.001	8.851	2.41	32.57	89
	1	25	88						
Angio-invasion	0	62	42	22.89	<0.001	17.31	3.76	79.62	89
	1	27	93						
Lymphocytes	0	95	53	6.98	0.01	0.16	0.34	0.78	108
	1	13	15						
Histiocytes	0	76	46	0.45	0.50	1.33	0.58	3.04	108
	1	32	53						
Eosinophils	0	82	42	6.2	0.013	3.18	1.24	8.15	108
	1	26	69						
Neutrophils	0	102	49	0.57	0.45	0.52	0.91	2.97	108
	1	6	33						
Plasma cells	0	75	39	9.00	0.003	3.65	1.52	8.76	108
	1	33	70						
Infiltration	0	96	52	5.84	0.16	0.16	0.04	0.89	108
	1	12	17						
Ulceration	0	87	43	2.83	0.09	2.48	0.84	7.31	104
	1	17	65						
Desmoplasia	0	66	21	47.09	<0.001	29.71	8.99	98.19	102
	1	36	89						
Margins	1	85	53	3.76	0.15	–	–	–	109
	2	15	40			0.59	0.19	1.81	
	3	9	22			0.25	0.05	1.29	

* ½ added to frequencies.

OR + odds ratio.

log likelihood χ^2^.

%META'S, %metastasis.

## Results

During the period 1982–2002, 915 SCCs of the skin (*n* = 852) and lip (*n* = 63) in 580 patients were found; 68 of these 915 tumours (7.4%) in 580 patients (11.72%) did metastasize. The prevalence of metastasis for lip SCC alone was 20.6% (13/63) and for skin tumours 6.5% (55/852). For both tumour locations 27% patients were women and 73% were men. The mean age among women was 79 years and 82 years for men. The mean follow-up was 5.7 years (0.25–21 years). In the total group three patients were immunosuppressed and seven had a recurrent lesion. Of the 68 metastases, 13 cases (19.1%) were on the lip and 37 (54.4%) were in the head and neck region. Nineteen (28%) of the metastases were located in the locoregional lymph nodes, mostly ipsilateral.

### Skin tumours (*n* = 110)

**Figure 1:**
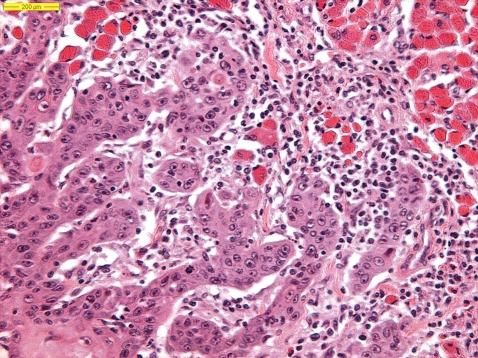
Clark level.

**Figure 2:**
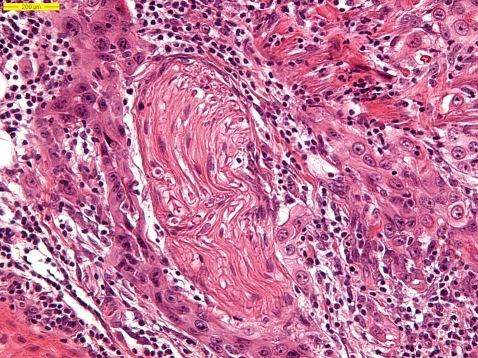
Perineural invasion.

**Figure 3:**
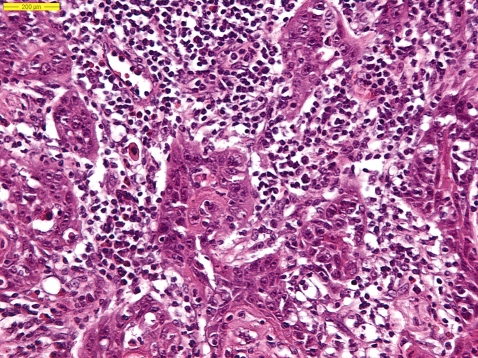
Plasma cells and eosinophils.

In the group of skin tumours 55 cases were compared with 55 controls. In the metastatic group of skin SCCs desmoplasia, Clark level (Figure 1), Breslow depth and maximum diameter, angio-invasion, grading, perineural invasion (Figure 2), plasma cells and eosinophils (Figure 3) proved to be statistically significantly related to metastasis. Table 1a lists the results of the histopathological characteristics of the skin tumour which metastasized.

In the multivariate analysis of skin SCC metastasis the eventual model found contains three risk factors and one protecting factor. In order of statistical importance the risk factors comprise: the maximum diameter of the tumour (in mm), having or not having tumour desmoplasia (Figures 5 and 6) and the reclassified, trichotomous Clark index. Finally, the only protective factor was found to be having or not having a lymphocytic infiltrate. Grade of differentiation was found to be the only remaining risk factor that was almost statistically significant (overall *P*-value of backward elimination χ^2^ was 0.083).

**Figure 4:**
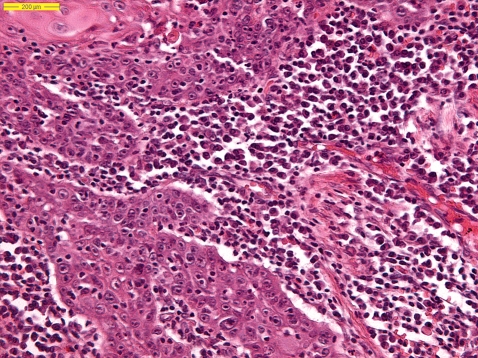
Plasma cells.

**Figure 5:**
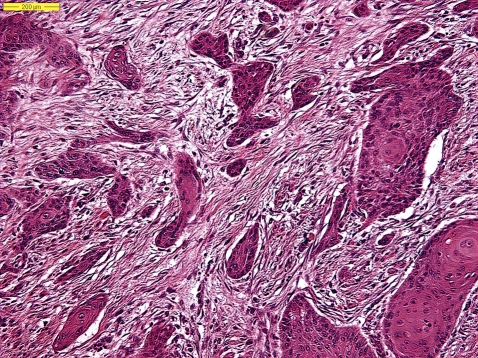
Desmoplasia.

**Figure 6:**
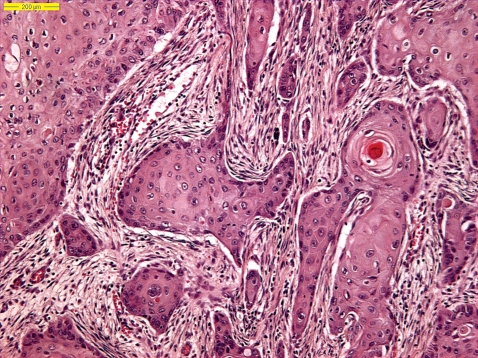
Desmoplasia.

### Lip tumours (*n* = 63)

In the group of lip tumours 13 cases were combined with 50 controls. In the group of lip SCCs, Breslow, plasma cells (Figure 4) and grading appeared to be statistically significant prognostic factors for metastasis. Table 1b lists the histopathological characteristics of the tumours of the lips. Of the tumours with Breslow thickness > 4.8 mm, 53% metastasized compared with 0% of SCCs < 4.8 mm. Breslow thickness was a statistically significant prognostic factor for metastasis [odds ratio (OR) = 3.71, *P* < 0.001].

**Table 1b:** Histopathological characteristics of the lips related to metastasis (*n* = 63)

		*n*	%META'S	X^2^L	*P*	OR	Lower	Upper	*n*
Grading	1	27	7	6.97	0.03				
	2	27	22			3.57	0.65	19.59	62
	3	8	50			12.50	1.69	92.25	
Max. diameter	≤15	28	18	0.64	0.42	1.70	0.46	6.21	54
	>15	26	27						
Breslow	≤4.8	19	0	16.89	<0.001	3.71*	0.77	6.65	37
	>4.8	18	53						
Perineural invasion	0	45	18	2.13	0.15	3.08	0.70	13.52	55
	1	10	40						
Angio-invasion	0	53	21	0.80	0.37	3.82	0.22	66.02	55
	1	2	50						
Lymphocytes	0	56	21	0.01	0.94	0.92	0.09	8.98	61
	1	5	20						
Histiocytes	0	37	19	0.32	0.57	1.43	0.42	4.92	61
	1	24	25						
Eosinophils	0	54	22	0.25	0.62	0.58	0.64	5.33	61
	1	7	14						
Neutrophils	0	57	21	0.33	0.86	1.25	0.12	13.12	61
	1	4	25						
Plasma cells	0	51	14	8.95	0.01	9.43	2.12	42.07	61
	1	10	60						
Infiltrationj	0	50	20	0.27	0.60	1.5	0.34	6.70	61
	1	11	27						
Ulceration	0	44	18	2.15	0.14	2.8	0.73	10.90	57
	1	13	39						
Desmoplasia	0	35	23	0.01	0.94	1.06	0.29	3.78	56
	1	21	21						

* ½ added to frequencies.

OR + odds ratio.

log likelihood χ^2^.

%META'S, %metastasis.

The multivariate analysis of lip SCC with metastasis showed that only one risk factor turned out to have a significant OR for metastasis, namely having or not having an infiltrate of plasma cells.

## Discussion

SCC of the skin is a potentially lethal form of skin cancer, responsible for 20% of all cutaneous malignancies. The incidence of metastasis from skin and lip SCCs varies between 0.5% and 16%.^18^ Some patients are known to die of local invasion of the tumour or metastases and these cases are difficult to detect in retrospective studies. Clinical and histopathological criteria have been proposed to identify tumours that may have an increased risk of metastasis.

Patients with metastatic disease have a poor prognosis, with 10-year survival rates of < 20%. It is therefore important to define high-risk tumours and treat these tumours aggressively with large excision margins and/or adjuvant radiotherapy, especially since the incidence of SCC of the skin is increasing. In our study we examined histopathological features that might predict the metastatic potential of SCCs of the skin and lip. To gain a better appreciation of histopathological parameters important in the development of metastases, we matched all cases. In our study we matched for the following factors: gender, primary or recurrent lesion, immunosuppression, location of the lesion, the therapy used and the length of follow-up. We realize that our study is retrospective and that for some characteristics relatively large numbers of values were missing. The group of patients with metastasis was small, so the statistical analysis of these results met with some difficulties. Future prospective studies should therefore be very large to interpret outcomes.

The study found that 68 of 915 tumours in 580 patients had metastasized. The rate of metastases was therefore 11.7% for the patients and 7.4% for the tumours. Males seem to have a higher incidence of metatastatic disease compared with women, even taking into account the larger numbers of SCCs in men.^19^ This was also found in our study. A review of the literature from 1940 until 1992 revealed that recurrent SCC has a significantly higher metastatic rate than primary SCC. For recurrent tumours there was a metastatic rate of 25% for skin SCC, a rate of 45% for ear SCC and a rate of > 30% for the lip. We found three (4.4%) recurrent lesions in the metastatic group.^11^

The effect of immunosuppression has been studied extensively, mostly because of the growing number of transplant recipients. It is not certain whether the higher number of metastatic lesions in an immunosuppressed patient is due to the more aggressive nature of every single SCC or the presence of multiple typical SCCs often seen in transplantation patients.^20^–^23^ In the group with metastasis studied, only one (1.5%) of the patients was immunosuppressed.

Tumour width, defined as the maximum diameter of the lesion, is an important prognostic factor for the metastatic potential of SCC. Significant correlation of tumour size with metastasis has been found in a number of reports.^8^,^11^,^12^,^15^,^16^,^18^,^24^ We also found tumour width to be significantly associated with metastatic potential. A size of ≥ 20 mm triples the risk of metastasis compared with lesions < 20 mm.^11^,^15^,^25^ In a German study, 78% of metastasizing tumours were > 20 mm.^13^

In our study 50% of the metastasized tumours were at least 15 mm in diameter. However, this does not mean smaller tumours are not capable of metastasis. In one study none of 14 metastasizing periauricular tumours was larger than 2 cm.^12^–^13^ This suggests that other prognostic factors should also be taken into account. Excision margins might also be important. Our results show that margins of < 2 mm are insufficient. This is in line with another study, which showed a cumulative tumour clearance of only 78% with 2-mm margins.^25^ This is far less than the 96% with 4-mm margins. Margins of 2 mm seemed to be insufficient in wider and less differentiated tumours.^25^

The depth of tumour invasion can be expressed according to Clark, or according to Breslow. Clark's classification is more widely used in the setting of SCC of the skin. As in our study, earlier studies point to the worse prognosis of deeper tumours.^11^,^12^,^15^,^16^,^26^–^28^

Some studies have found metastases only when there was invasion of subcutaneous tissue. This was not the case in our population, although the tumours were at least Clark II. Breuniger and colleagues found a metastatic rate of < 1% when only the dermis was invaded, a rate of > 4% with invasion of the subcutaneous fat and a rate of > 12% when deeper tissue was invaded. Measuring Breslow depth (vertical tumour thickness) is a more time-consuming way of expressing tumour depth. In our study all metastatic ‘pure’ skin tumours were > 2.85 mm thick. SCC of the skin of < 2.85 mm probably does not metastasize at all.^12^,^15^–^17^,^24^,^26^,^28^–^30^

In our study, measurements of tumour differentiation were based on cellular polymorphism and (de)squamation. Both are significantly associated with metastatic potential according to Petter and Haustein.^12^ Like most studies, we did not differentiate the histological grading further with regard to these separate factors. A significant relationship between differentiation and metastases has often been found, although some studies could not establish such a relationship.^1^,^14^,^19^,^24^,^29^,^31^,^32^ Our results confirm it and endorse the importance of tumour differentiation. Ulceration, histiocytic inflammation and neutrophilic inflammation were not related to metastasis in our patients. Some previous studies are in agreement with our own^8^,^18^,^24^,^30^,^33^,^34^ but other studies have reported that these factors are related to metastases.^11^,^12^,^16^,^32^

The incidence of perineural invasion in SCC is reported to be 3.7%.^11^ Lesions with perineural invasion have a significantly higher risk of metastatic disease. Metastases in neurotropic skin SCC occur in 5.9–34.8%, depending on treatment regimen. In our study perineural invasion also seems to be an important factor in skin metastasis. Lip SCC has an even worse prognosis when neurotropism is present, with reported metastatic rates of 60–80%. We did not confirm this in our study.

Angio-invasion is, to our knowledge, a factor less extensively investigated.^30^ Our results have shown a statistically significant relationship with metastasis in this respect.

Desmoplastic SCC has been defined as a SCC with fine, diffusely infiltrating branches of tumour cells at the periphery, surrounded by a strong fibrotic stromal reaction. Desmoplastic SCC frequently metastasizes.^12^,^16^,^23^ We did find many tumours that matched the criteria for desmoplastic SCC. However, we saw at least a little desmoplasia in every metastasizing tumour. Based on the results of our study, desmoplasia might therefore be regarded as an important factor in SCC of the skin.

## Conclusion

A typical metastatic SCC showed: a tumour width of at least 15 mm, a vertical tumour thickness of at least 2 mm and at least Clark II, less differentiation, presence of desmoplasia and invasion with eosinophils and plasma cells.

The next step will be to establish a prospective study with a follow-up of at least 3 years, in order to establish a risk profile of SCC of the skin, based on histopathological tumour characteristics.

## Abbreviation

SCC: squamous cell carcinoma
